# Labour Market Participation of Older Workers: Drivers and Obstacles

**DOI:** 10.1007/s10272-021-1010-9

**Published:** 2021-12-11

**Authors:** Ulrich Walwei, Jürgen Deller

**Affiliations:** 1 Institute for Employment Research (IAB), German Federal Employment Agency (BA), Regensburger Strasse 104, 90478 Nürnberg, Germany; 2 Institute of Management & Organization, Leuphana University of Lueneburg, Universitätsallee 1, 21335 Lüneburg, Germany

## Abstract

From an international comparative point of view the paper deals with driving forces and potential obstacles for the labour market participation of older workers. It goes into depth by focusing on four case studies that seem to be typical for different contexts. Germany, Israel, Italy and Sweden were selected in order to examine the development and the situation of older workers in detail. Each country stands for a specific configuration, e.g. because it may represent a trend reversal, a continuously outstanding performance, or lasting problems. The cases also provde information on pension reforms and approaches to better manage ageing workforces, in some cases including a new balance of work and retirement. Being aware of the different country situations, it becomes obvious that one size of politics does not fit all. Independent of national policies, employability over the life cycle should gain more attention. Regarding future developments, continuous skill improvement and a healthy work environment are indispensable to keep older workers in work.

Demographic change is a great challenge for the economy as well as the labour market. It is likely that the population in most industrialised countries will grow slower or even shrink in due course, and so will the labour force. Additionally, people and workers are generally growing older. The process of ageing causes numerous questions for economic systems. What does it mean for economic development and trends in labour productivity in the long term? To what extent can implicit knowledge be maintained and skill shortages be avoided? Are social security systems based on contributions by workers sustainable?

A key factor is the employment-to-population rate of workers, particularly of older workers. How far this growing part of the workforce can be utilised is of particular relevance to the future size of labour supply in a given country. A labour force that grows slower or even shrinks has the potential to reduce economic growth and income opportunities for older workers. The more older workers are employed, the more they contribute to economic activities, facilitate knowledge transfer, offer valuable skills, relieve the burden on social security systems and generate their own income.

From a comparative point of view, this paper deals with the driving forces of employment-to-population rates of older workers. The comparison particularly focusses on the process of ageing in European workforces. The paper consists of four case studies that are particularly typical in different contexts: Germany, Israel, Italy and Sweden were selected to examine the development and the situation of older workers in detail. Each country stands for a specific configuration, e.g. because it may represent a trend reversal, a continuously outstanding performance or unsolved problems. The conclusion summarises the main findings and gives tentative recommendations for labour market policies.

## Labour market participation of older workers

Due to the differences in employment rates of older workers over time and between countries and their great significance for future developments, a major question is what may drive the evolution of these ratios. Employment rates of older workers can be influenced by three main factors (Walwei, 2018a). First, differences in the labour

* Both authors contributed equally to this manuscript.

**Ulrich Walwei**, Institute for Employment Research (IAB), Nuremberg, Germany.

**Jürgen Deller**, Leuphana University of Lueneburg, Germany.

market situation and economic development of a country are of relevance. A favourable labour market development improves the outlook of all groups of workers, including older workers. This argument can perfectly be illustrated with Kennedy's (1963) famous phrase that "a rising tide lifts all boats". Secondly, changes in characteristics of jobs and workers may also play an important role. Modified job requirements, differences in labour market participation by gender and educational attainment, and variations in the health situation of the population can be associated with the labour market participation of older workers. Thirdly, institutional reforms may create incentives or disincentives for older workers to participate in the labour market. Regulations dealing with pension schemes and unemployment benefits as long as older persons are concerned are of particular importance.

The case studies of Germany, Israel, Italy and Sweden present different cultural backgrounds and typical developments in industrialised countries. In addition, all of them represent different welfare state models.

Table 1
**Ageing of population and workforce**

| | | Germany | Israel | Italy | Sweden | OECD |
|---|---|---|---|---|---|---|
| Median age of total population (years) | | | | | | |
| 2018 | | 46.0 | 29.8 | 46.3 | 40.6 | 39.8 |
| Total employment rates by age groups (%) | | | | | | |
| 1993 | 25-54 | 76.8 | 68.9 | 66.7 | 83.2 | 74.4 |
| | 55-64 | 35.9 | 45.2 | 30.4 | 63.4 | 46.1 |
| 2018 | 25-54 | 84.9 | 80.1 | 69.8 | 86.6 | 78.4 |
| | 55-64 | 71.5 | 67.3 | 53.7 | 78.2 | 61.4 |
| Employment rates by gender, age group 55-64 (%) | | | | | | |
| 1993 | Male | 47.8 | 62.2 | 48.2 | 65.9 | 59.7 |
| | Female | 24.2 | 29.6 | 14.1 | 60.9 | 33.4 |
| 2018 | Male | 76.1 | 73.7 | 64.2 | 80.5 | 70.2 |
| | Female | 66.9 | 61.2 | 43.9 | 75.8 | 53.1 |
| Employment rates, age group 65+ (%) | | | | | | |
| 2018 | | 7.4 | 21.9 | 4.7 | 16.9 | 15.1 |

Sources: United Nations (2019); OECD (2021).

## Four country case studies

Germany has been chosen for its trend reversal regarding the labour market integration of older workers; Sweden for its consistently high participation rate of older workers; Italy for its difficulties with achieving a high labour market participation of older workers; and Israel for its rather young population as an interesting counter model. Table 1 illustrates the differences between the four countries. It indicates that in 2018 the employment rate of older workers (55 to 64 years) was highest in Sweden for both men and women. The largest increase of the employment-to-population rate of older workers between 1993 and 2018 can be observed in Germany, while Italy shows the lowest increase. Israel has by far the youngest population – as measured by the median age – and the highest share of workers older than 65 years.

The following case studies do not aim at providing any causal explanation for developments of employment-to-population ratios of older workers in the particular countries. Instead, they offer circumstantial evidence in order to illustrate which factors may have been of importance for varying developments.

### Germany

Before entering the COVID-19 pandemic, the German labour market was in rather good shape (Bauer et al., 2020; Schneider and Rinne, 2019). While employment-to-population ratios increased across the board since the mid-2000s, the increase has been particularly strong among older workers. Moreover, there is clear evidence that older workers profited from the recent employment boom (Walwei, 2018a). Like several other European countries, Germany is confronted with continuous population ageing. The labour market situation of older workers was poor compared to those in other countries and younger workers until the early 1990s. This applies even more to women than to men. In recent years, however, the labour market participation of older workers has improved considerably. The positive development of the employment-to-population rate of older workers is associated with the higher stability of already existing employment relationships, i.e. one can observe a lower exit rate of older workers (Dietz and Walwei, 2011). The positive trend refers not only to workers aged 55 to 64 who are still part of the workforce but also to those who are already retired (Anger et al., 2018; Walwei, 2018b). Due to increasing skill shortages, firms have made great efforts to retain workers who were eligible for pensions (Czepek et al., 2017). Further evidence shows that a stable employment biography before entering the retirement age favours the likelihood of labour market participation of retired persons (Westermeier, 2019).

Nevertheless, the older workers are, the lower their labour market participation (Walwei, 2018b). Especially if older persons are already unemployed, they experience severe difficulties (re-)entering the labour market (Dietz and Walwei, 2011). The potential devaluation of marketable competencies over time, reservations of employers regarding their flexibility and resilience, and the potential of elevated health risks of older people are a few of the factors that may explain the low entry rates of older workers.

The positive trend of an increased labour market participation of older workers started long before the beginning of the boom period in 2005. One contributing factor relates to the skill level of workers, which has significantly increased over time (Bosch, 2011). Studies indicate a positive relationship between the skill level of workers and their employment-to-population ratio independent of their particular age (Dietz and Walwei, 2011). This means that being advanced in age does not necessarily translate into lower chances of being employed. However, the combination of higher age and lower skills markedly deteriorates the chance of being employed.

Further evidence reveals that the improved labour market performance of older workers relies to a considerable extent on higher employment-to-population ratios of women (Walwei, 2018b). This implies that each female worker entering the old age level incorporates a stronger labour market affinity and pushes the labour market participation of older workers. The favourable development of health over time also plays an important role. Regular reports indicate an improved health of the German population over time (Gesundheitsberichterstattung des Bundes, 2020). Since the 1990s work accidents decreased by one half. In addition, the EU-SILC-survey indicates for Germany a steady improvement in self-assessed health, particularly for the group aged 50 to 65 years.

In Germany, a number of relevant institutional changes have been implemented in the 1990s and at the beginning of the new millennium. They have considerably increased work incentives for older workers (Dietz and Walwei, 2011; Steiner, 2017). Already at the end of the 1990s, the retirement age for unemployed, women, disabled and long-term insured[1] increased. In 2008, the general retirement age went from 65 to 67 years. In 2014, workers who were 63 years or older were offered the opportunity to receive their pensions earlier without any penalty. Although this new option has been frequently used, it should not be seen as a substantial rollback of retirement policies in Germany (Börsch-Supan et al., 2014). Institutional changes also put more pressure on unemployed in general by activating people instead of financing unemployment. The so-called Hartz reforms introduced a means-tested tax-based unemployment benefit II complementary to the contribution-based unemployment benefit I. This level of social protection became less generous, especially for long-term unemployed. Additionally, the maximum duration of entitlement to regular unemployment benefit I for older workers was reduced from 32 months to 24 months. Findings indicate that these reforms have contributed to a stronger individual search intensity and a greater willingness of the unemployed to accept less attractive jobs (Eichhorst and Marx, 2011; Möller, 2015; Walwei, 2015).

1 At least 45 years of qualifying pension contributions.

### Israel

Israel is still a young country making it particularly interesting.[2] Israel will unavoidably age in the future. However, even in 2050, it will remain comparatively young, with the smallest age group of 65 years or older of all countries in focus (OECD, 2021; Deller and Walwei, forthcoming). Israel's youngest age group will become slightly smaller: In 2018, it was about one-third of the population, and more than 20% will belong to this group in 2050.

According to Larom and Lifshitz (2018), the Israeli labour market was performing well before entering the COVID-19 pandemic. It was characterised by high employment, low unemployment and increasing hourly wages. Nevertheless, two population subgroups, the Arab population (20% of Israel's total population) and the ultra-Orthodox (Haredi) Jewish population (10%) still lag behind today's majority group (non-Orthodox Jews). This is true for both employment and earnings. However, in the upcoming decades the proportions will shift and, together, Arab and Haredi will represent half of the total population (Larom and Lifshitz, 2018, 11). In order to continue the positive labour market development, it seems necessary to further increase the employment rates among these groups.

Israel's employment rate increased by ten percentage points during the last 15 years, hardly affected by the global financial crisis. Axelrad (2020) reports an overall labour force participation rate of 19.5% for individuals aged 65 and older compared to 10.4% in 2004. These higher rates make Israel second only to Denmark compared to 20 European countries (Axelrad, 2018). They may result from the increased retirement age, from immigrants without pension plans and entitlements or only low social security retirement benefits (Axelrad, 2020). Unemployment has followed a reversal trend. In 2000, the unemployment rate in Israel was around 10%. Since 2004, it has declined rapidly, virtually without a gender gap, to about 4% by 2015.

The high-tech sector employs about 8%-9% of Israel's workers (as of 2017; Fuchs and Weiss, 2018) and is highly relevant for Israel's exports (42%). Employment rates dif-

2 In addition to others, this case study especially uses the works of Fuchs and Weiss (2018) as well as Larom and Lifshitz (2018). This literature is well suited for further readings.

fer by sex and across population groups. By 2015, the overall male employment rate had risen to 81.4%, slightly below the OECD average. The change among women was even more noticeable. By 2015, the employment rate among women in Israel reached 72%, 7.8 percentage points higher than the OECD average. The employment gap between men and women in Israel was only 9.4 percentage points in 2015 (compared to 17.9 percentage points in OECD countries). The data on the participation rates show a similar picture.

The increase in employment was accompanied by higher wages and household incomes. Although individuals who are low-skilled, older and from large families experienced the fastest growth in employment, significant gaps still exist, with especially low employment among Arab women and Haredi men.

The change in employment is largely due to a series of policy measures. Some have increased the incentive to work, while others have cut unemployment benefits. In 1990, Israel improved access to higher education. A much higher proportion of the population now has a college degree. The share of college students in a cohort reached 50% in 2013, higher than in the OECD as a whole. According to Larom and Lifshitz (2018), 30% of the increase in employment can be attributed to the educational attainment.

Retirement age is characterised by a gender gap. This gap is not expected to close. In 2004, the mandatory retirement age was raised from 65 to 67 for men and from 60 to 62 for women. For over a decade, the government has been trying to raise women's retirement age to 64, without success however (Fuchs and Weiss, 2018). The higher retirement age had a direct effect on employment. Compared to other age groups, the 55-64 age group has the lowest employment rate but the fastest increase in employment. As the employment of both Arab and Haredi women continues to have low rates in the 55-64 age group, there would have to be a behavioural change to realise more participation in the labour market. As a result, a discussion has commenced about the importance of support for these groups, e.g. through higher education and designated training centres.

Reforms of Israel's welfare and benefit system since 2002 have tried to increase the incentive to work and to decrease the benefits of non-employment. The level of both the unemployment benefits and period of entitlement were reduced (Larom and Lifshitz, 2018). These changes substantially reduced the number of eligible individuals. Following these reforms, the levels of income support, child allowance and entitlement were also cut dramatically. The reform lowered the payment per child and disconnected it from the number of children in the family. The effect was positive on employment, but negative on fertility.

## Italy

The case of Italy[3] is quite similar to Germany in some respects, e.g. continuous ageing as well as a substantial increase in the labour force participation of older workers (aged 55-64). It differs, however, in others ways, e.g. the lower labour force participation of younger individuals (aged 15-24). The share of the elderly population (65 and over) as part of the total population increased from 1988 to 2018 by 8.5 percentage points, while the working age population (15-64 years) shrank. According to Socci et al. (2017), Italy needs a "New Deal" among all stakeholders to develop a national strategy for older workers.

Italy is one of the largest economies in Europe. Its labour market is characterised by differences across age groups, including high youth unemployment. Another key challenge is a north-south disparity with the north continuing to be more dynamic (Marino and Nunziata, 2017). Recently, the share of inactive workers in the Italian labour market increased, and the labour market shrank (Colussi, 2020). The youth (age 15-24) face high unemployment and low participation rates, which decreased between 2000 and 2016 by 15 percentage points (Marino and Nunziata, 2017). Only about a quarter of this group is working. This is also due to the emergence of the NEET youth (those not in education, employment, or training), which totals about one-fifth of the age group. Additionally, the undeclared employment level is high. Since 2009, job vacancies have increased. Italy has experienced a moderate recovery. The large increase of youth unemployment, however, remains a major problem.

Since 2000, older workers' (aged 55-64) labour market participation has accelerated and increased substantially after the 2011 "Fornero" pension reforms. There was an increase of 25 percentage points between 2000 and 2016, standing at 53.4% in 2016 (Marino and Nunziata, 2017).

Although female labour force participation is still low, it has increased by five percentage points from 2000 to 2016. There is a large variation between regions, with specific regions in the north and south alike being above the median of the increase in participation. The low female participation rate can be explained by institutional characteristics including a lack of childcare options and

3 In additon to others, this case study especially uses the works of Marino and Nunziata (2017) as well as OECD (2018). This literature is well suited for further readings.

of flexible working arrangements. The female participation rate ranges from 60% to 67% in the north and 37% to 53% in the south. At the same time, male participation in the labour force remained quite stable. Real earnings have increased; however, productivity remains at relatively low levels compared to other European countries.

Employment policies for older workers in Italy are partly drafted by the regions, not the national government (Socci et al., 2017). The OECD (2018) identified Italy's tactics to promote longer working life as raising the statutory age of retirement, enhancing participation in training and strengthening workplace health and safety as well as targeting workers at risk of unemployment in some regions. Three core policy areas are mentioned: rewarding work and later retirement, encouraging employers to retain and hire older workers, and promoting the employability of workers throughout their working lives.

Italy tried to reward work and later retirement in three different ways. First, by enhancing incentives to continue working at an older age. In 1995, the Italian pension system switched from a defined benefit pension scheme to a notional defined contribution scheme. The "Fornero" pension reform in 2011 accelerated this transition in a more "coercive way" (Socci et al., 2017). At retirement, the accumulated notional capital is converted into an annuity, taking average life expectancy at retirement into account. The second tier is an income-tested "old-age social allowance" entitling employees to a severance payment benefit. Private pension plans form the third tier. In 2012, the retirement age for men was set between 66 and 70 years of age, the respective age for women was set at 62 to be raised to 66. There are incentives for employees to continue working as the pension level increases if the person postpones retirement. Since 2008, it is possible to combine pension income, including early retirement pensions, with income from self-employment or project work. The percentage of retirees working after the age of 65 is very low. Those who do work either have low skill profiles or are highly skilled professionals.

Second, pension reforms restricted the use of early retirement schemes. A new early retirement programme was introduced to have a positive effect on the entry of young workers into the labour market. This effect, however, is widely discussed as the impact is likely to be low over the long term (Bertoni and Brunello, 2017). Workers in arduous and hazardous jobs can access early retirement pensions more easily. Third, different measures to prevent welfare benefits from being used as alternative pathways to early retirement have been introduced. Overall, active ageing has only been developed quite recently in Italy (Socci et al., 2017).

### Sweden

Sweden is one of the European countries with the highest population growth over the last few decades. Besides a considerable positive net migration, the fertility rate (1.85 children per woman) is closer to the population replacement level than in most other European countries.

Although overall employment rates in Sweden were already comparatively high in the late 1980s, they increased even further for almost all groups of workers in recent decades. They are still among the highest in the European Union. The economic crisis in the early 1990s brought about a significant increase in unemployment and a temporary decrease in labour force participation (OECD, 2020). Nowadays, unemployment in Sweden is lower than the EU average (Eurostat, 2020). Overall, the 2008 financial crisis had small negative impacts on the Swedish labour market (Albin et al., 2015). Nevertheless, the unemployment rate in Sweden is currently more than twice as high as it was in the 1970s and 1980s. Workers without upper secondary school education do poorly in the Swedish labour market, and their problems appear to be growing.

The high female labour market participation is one of the major explanations for steadily high employment-to-population rates for all groups of workers, but especially for older workers. The labour market participation of women increased particularly until the 1990s when a "feminisation of the labour force" took place (Albin et al., 2015). "Housewives" have more or less disappeared in Swedish society. Nevertheless, the part-time employment rates of women are still much higher compared to men (OECD, 2020). The employment rate among older workers is comparatively high and still trending up (Laun and Palme, 2018). Recently, even the 65-69 age group showed an increase in employment-to-population ratios.

Structural change is also of relevance for transitions between work and retirement because certain industries and their particular jobs might be affected by the transformation more than other industries and corresponding jobs. One can identify a clear socio-economic gradient in the age of retirement: Low-skilled jobs have lower wages and worse working conditions, working environments and occupation protection (Laun and Palme, 2018). Although the incidence of long-term unemployment as part of total unemployment is higher for older workers than for the so-called "best agers" (25 to 54 years), the level is still rather low compared to other countries.

The comparatively high employment-to-population ratio was not only favoured by labour market developments, but also by changes in characteristics of workers and jobs. Circumstantial evidence addresses four influential factors. First, failing health has been identified as the most common reason for retirement (Albin et al., 2015). Overall, statistics indicate that the population in Sweden shows improved health over time. Particularly between the 1960s and 1980s, mortality decreased and surveys report a steady improvement in self-assessed health, especially regarding older men (Laun and Palme, 2018). Research shows that good mental and physical work environments potentially avoid high-risk jobs, physical exposure and disability. Second, education in Sweden is an important gradient for retirement age. In this respect, one can observe significant changes in educational attainment in Sweden. Current cohorts of older workers are more educated than previous ones and tend to retire later (Venti and Wise, 2015). Third, the retirement age is also associated with retirement decisions by life partners or close friends (Laun and Palme, 2018). Couples' joint decision-making leads to an earlier exit for women and later exits for men. Fourth, regarding work requirements there are hints in Sweden that jobs are getting less demanding, e.g. the number of deaths due to work accidents has decreased. Self-reported assessments indicate lower physical demands (Laun and Palme, 2018).

In the 1980s, worries about the viability of pension systems started against the backdrop of an ageing population and lower economic growth. Several political decisions were taken from the early 1990s onwards to counteract early retirement and increase the actual retirement age (Laun and Wallenius, 2015). They aimed at increasing work incentives, influencing retirement behaviour and, as a consequence, delaying exit from the labour force. Such reforms consisted of several elements. The level of pensions is now more proportional to contributions. A payroll tax reduction for retired persons was implemented, combined with a reduction of the income tax. This regulation contributed to the delayed labour force exit, which also has helped to keep employment-to-population rates of older workers high (Laun and Palme, 2018). The increase of the mandatory retirement age was not so relevant for persons aged 64 or younger but more so for the 65-69 age group. However, the strongest effect on persistently high employment-to-population rates of older workers can be attributed to stricter rules for the disability insurance programme.

## Conclusions

This comparative paper reflects drivers and obstacles of labour market participation of workers with a specific focus on older workers in four country cases. Each case stands for a specific situation, as well as for specific regulations and changes. Additionally, the cases include information on pension reforms and approaches to better manage ageing workforces, in some cases including a new balance of work and retirement.

In Germany, one can observe a longer-lasting trend of an increasing employment-to-population ratio of older workers. The economic upswing vitalised the labour market in general with a rather positive effect on older workers. However, the situation was already improving in times of weak economic and employment growth – thus, it is not only growth-induced. Pension and labour market reforms can claim a considerable share of success for increasing the number of older workers (Steiner, 2017). Other issues such as a growing educational attainment and increasing labour force participation also seem to play an important role.

Israel is a young country, and its labour market has improved considerably. A series of successful policy measures have laid the foundation. Following a period of exceptionally rapid increase, the employment rate went up more than ten percentage points and unemployment has fallen to 4%. However, serious challenges face two minority groups: Arabs and Haredi Jews. They lag behind in both employment and earnings. While men and women have different legal retirement ages, this finding is highly interesting: Regarding early versus later retirement, Israel has both a strong group of individuals leaving early and another strong group leaving late, thus creating an interesting research case.

Italy has a challenge employing age groups equally. The labour market is characterised by growing participation of the oldest age group while the youngest has major difficulties entering the labour market, partially due to the high share of NEET youth. The second challenge is to continue the development equally in both the north and south. It is necessary to strengthen this. Italy is also a good example of demographically driven pension reforms leading to behavioural change. However, they may also contribute to unbalanced groups in the labour market. The importance of education is demonstrated by the persistant problem of NEET youth. Shaping this development successfully may set an example for other countries as well.

Sweden has one of the highest labour force participation rates among older workers, driven to a large extent by high female labour market participation. There is evidence that retirement policies may have led to delayed labour market exit, mainly through stricter rules for disability eligibility. Exit patterns from employment to retirement have become increasingly heterogeneous. They augment inequalities between older people in retirement, particularly regarding educational attainment. Findings for Sweden suggest that good health and high education are the most important factors for a long career and late retirement. It is argued that policies should focus more on providing education and training to vulnerable groups (Albin et al., 2015).

Given the different country situations, we have not looked for best practices but for design options. Some aspects are similar, others very different. Case studies show that it is important to understand the mechanisms behind different developments. A comparatively young country like Israel may have advantages from a constant inflow of young and well-educated workers. At the same time, it faces challenges like the integration of diverse population groups into the labour market, groups that so far have shown very different labour market behaviours. Compared to Israel, the situations in Germany, Italy and Sweden are quite different. Thus, being aware of the country situations, it becomes obvious that one size of politics does not fit all. Changes must be tailor-made.

The current COVID-19 pandemic may have various consequences for older workers. The reluctance to recruit older workers may be enforced in the current pandemic, when employers may have additional concerns about their health risks. Flexible work is often seen as a suitable way to encourage older individuals to remain in workforce longer, e.g. by facilitating working from home. Digitisation has been pushed by the pandemic, implying an increased need for new capabilities of workers. Therefore, employability over the life cycle should gain more attention. Continuous skill improvement and a healthy work environment are indispensable to keep older workers in work.
